# CLINICIAN EXPRESSIONS OF CONDOLENCE AFTER THE DEATH OF A PATIENT: AN INTERNATIONAL SURVEY

**DOI:** 10.1093/geroni/igad104.2365

**Published:** 2023-12-21

**Authors:** Katlynn Van Ogtrop, Sofie Nelson, Christian Nouryan, Kevin Carratu, Maria Carney, MD

**Affiliations:** Northwell Health, Manhasset, New York, United States; Northwell Health Physician Partners Geriatrics and Palliative Medicine at New Hyde Park, New Hyde Park, New York, United States; Northwell Health, Manhasset, New York, United States; Feinstein Institute for Medical Research, Manhasset, New York, United States; Northwell Health, Manhasset, New York, United States

## Abstract

There is little literature about expressions of condolence from providers to family members of those that have died, and no known literature reported during the COVID-19 pandemic. This study utilized an investigator-developed survey of healthcare clinicians about contacting families of patients who have died. It conducted via email and online from October to December 2021. Of 131 respondents, 67% were female, from 23 states, Canada, and UK. Half (49%) had >15 years experience, and most (85%) were attending physicians. The majority (99%) reported that a patient had died in their care within last year, while 18% reported lost >10 patients per month. Methods of condolences were phone calls, and personal letters. Most (67%) reported no change in contacting families, while 23% increased. When asked if they would be interested in education on expressing condolences, 47% responded “yes.” Barriers to condolences were time (64%), unsure of what to say (20%), afraid family will be upset (19%), medical/legal reasons (10%), no training (8%), and no personal relationship (7%). Most, (85%) reported that most calls “went well or better than expected.” Females (vs. male) reported often/always sending letters (45% vs 20%, p=0.13), and often/always calling by phone significantly more (71% vs 63%, p=0.04). Younger (< 40) clinicians (vs. older) reported being very/moderately comfortable talking to families (72% vs 78%, p=0.79), phone calls (64% vs 69%, p=0.68) and personal letters (27% vs 42%, p=0.91). Nearly half of respondents requested training. This is a practice that should be further studied for clinician and family experience purposes.

